# Three-Dimensional ResNeXt Network Using Feature Fusion and Label Smoothing for Hyperspectral Image Classification

**DOI:** 10.3390/s20061652

**Published:** 2020-03-16

**Authors:** Peida Wu, Ziguan Cui, Zongliang Gan, Feng Liu

**Affiliations:** College of Telecommunications and Information Engineering, Nanjing University of Posts and Telecommunications, Nanjing 210003, China; 1218012329@njupt.edu.cn (P.W.); ganzongliang@gmail.com (Z.G.); liuf@njupt.edu.cn (F.L.)

**Keywords:** deep learning, hyperspectral image classification, group convolution, spectral-spatial features

## Abstract

In recent years, deep learning methods have been widely used in the hyperspectral image (HSI) classification tasks. Among them, spectral-spatial combined methods based on the three-dimensional (3-D) convolution have shown good performance. However, because of the three-dimensional convolution, increasing network depth will result in a dramatic rise in the number of parameters. In addition, the previous methods do not make full use of spectral information. They mostly use the data after dimensionality reduction directly as the input of networks, which result in poor classification ability in some categories with small numbers of samples. To address the above two issues, in this paper, we designed an end-to-end 3D-ResNeXt network which adopts feature fusion and label smoothing strategy further. On the one hand, the residual connections and split-transform-merge strategy can alleviate the declining-accuracy phenomenon and decrease the number of parameters. We can adjust the hyperparameter cardinality instead of the network depth to extract more discriminative features of HSIs and improve the classification accuracy. On the other hand, in order to improve the classification accuracies of classes with small numbers of samples, we enrich the input of the 3D-ResNeXt spectral-spatial feature learning network by additional spectral feature learning, and finally use a loss function modified by label smoothing strategy to solve the imbalance of classes. The experimental results on three popular HSI datasets demonstrate the superiority of our proposed network and an effective improvement in the accuracies especially for the classes with small numbers of training samples.

## 1. Introduction

With the rapid development of hyperspectral imaging technology, the increasing number and the higher quality of available hyperspectral data make hyperspectral image processing a critical technique in numerous practical applications, such as vegetation ecology [1], atmosphere science [1], geology and mineral resources [2,3], ocean research [4], and precision agriculture [5]. Hyperspectral Images (HSIs) are characterized by their abundant spectral features, which enhance the accuracy of identifying the corresponding ground materials [6]. Though HSIs take the form of a data cube containing both spectral and spatial information, compared to the high spectral resolution, the spatial resolution of HSIs is very low, which can only provide less information of the geometric relationship of image pixels to each other. In addition, owing to the increasing variety of ground materials, which leads to the complexity of spectral information, and the scarcity of samples that have been labeled in the experimentations, HSI classification tasks, especially the tasks to improve the classification accuracy of classes with small samples, have become more challenging.

Traditional machine learning (ML) based pixelwise HSI classification methods mainly consist of two steps: feature engineering and classifier training [7]. First, feature engineering methods are used to reduce the high dimensionality of HSI pixels, and then extract the most representative features or select informative spectral bands [8]. Second, these selected features from the first step are trained in the classifiers through nonlinear transformation [9]. Among those classifiers, support vector machine (SVM) is the most widely used one for HSI classification tasks [10,11,12,13]. For instance, Reference [12] adopted SVM as the final classifier after a reduction of the dimensionality. Tensor-based classification models, considering a rank-1 canonical decomposition of weight parameters [14], were applied to the HSI classification task, where a limited number of samples are available. References [14,15] introduced a rank-1 feedforward neural network (FNN) nonlinear classifier instead of SVM, and they yielded better results than traditional deep learning methods when only a small number of training samples is available. But usually in the feature engineering, inappropriate dimensionality reduction in the spectral domain may lead to the loss of much of the spectral information. In other words, it does not fully utilize the spectral information. In summary, traditional ML based methods usually cannot get the most effective feature expression for the ground materials, and the design of the feature extraction methods is very difficult because it relies on the experience of experts. Therefore, the classification accuracy of traditional ML based methods is low relatively.

In recent decades, deep learning has made considerable progress in hyperspectral image classification tasks. Compared with traditional machine learning methods, deep learning network can be trained automatically to extract more abstract and task relevant features with fewer artificial constraints [16,17,18,19,20,21,22]. The end-to-end approach reduces the possibility of the information loss in pre-processing dimensionality reduction. For example, Reference [16] introduced a deep belief network (DBN) and used a logistic regression layer to classify hyperspectral sensor data. Though it has stronger ability of feature extraction than traditional classifiers, its learning process is slow and improper parameter selection may result in a local optimal solution. Reference [17] firstly used principal components analysis (PCA) to reduce the dimensions of raw input HSI data, and then constructed the spectral-spatial features by convolutional neural network. The classification task was conducted by a multi-layer perceptron (MLP). However, non-principal components with small variance may also contain important information, so the information loss caused by PCA algorithm may lead to poor classification performance. The stacked autoencoders (SAEs), based on multilayer neural networks, were used as feature extractors to capture the representative stacked spectral and spatial features [22]. However, the input data of SAEs must be flattened to one-dimensional vectors, leading to the flatten training samples losing the spatial information. In addition, SAE is an unsupervised deep learning method, which cannot fully utilize label information.

The convolutional neural network (CNN) mimics the way of information transition of biological neurons and shows outstanding performance in image processing especially image classification tasks. And several representative works based on CNN have been proposed to improve HSI classification performance [23]. For example, a CNN-based feature extractor was proposed in Reference [24], which could learn discriminative representations from pixel pairs by 2-D CNNs and use a voting strategy to smooth final classification maps [24]. Reference [25] proposed a feature-learning CNN (FL-CNN) to learn spectral and spatial features. In the FL-CNN network, both spatial context and spectral information are integrated into the framework [25]. Considering that HSIs have both spatial and spectral information, Reference [26] divided the network into spectral and spatial two consecutive learning blocks, which means that 2-D CNNs were used to independently extract the features of these two domains. A contextual deep fully convolutional neural (FCN) network [27] was proposed to exploiting local spectral-spatial information and predict the corresponding label of each pixel vector. The spatial and spectral feature maps can be obtained from the multi-scale filters based on 2-D CNNs, and then are combined together to form a joint spectral-spatial feature map to feed the fully convolutional network. To obtain a remarkable reduction of computational cost, spectral-spatial dual-channel dense network (SSDC-DenseNet) was proposed to utilize 2D filters to replace 3D ones for model scale reduction [28]. The architecture of Reference [29] consists of a hybrid of 1D and 2D convolutional layers based on capsule network to extract spatial and spectral feature respectively. Reference [30] adopted an organic combination of 3D-2D-CNN, residual learning, and depth-separable convolutions to learn spectral-spatial features. In Reference [31], a balanced local discriminant embedding algorithm was proposed to extract spectral features, and the 2-D CNN-based spatial information combined with these spectral features was sent to the classifier for HSI classification. However, due to the imbalance of the HSI dataset, much information, especially those with a small number of training samples, may be lost during the spatial feature extraction process, which means that 2D-CNN cannot fully utilize the spectral and spatial information at the same time.

Therefore, three-dimensional convolution neural networks (3-D CNNs) were adopted to extract deep spectral-spatial features directly from raw HSIs and delivered promising classification outcomes [23]. By applying 3D kernels to 3D HSI data cubes, Reference [32] introduced 3-D CNNs into HSI classification tasks to extract the spectral features and the spatial feature simultaneously. This approach took full advantage of the structural characteristics of the 3D HSI data. Reference [33] applied 3-D CNN to learn hierarchical multi-scale spectral-spatial features which could generate multi-scale image patches for each pixel at different spatial scales. But when the network becomes deeper, it may be difficult for the 3D-CNN models to further improve the classification accuracy. Therefore, Reference [34] adopted the residual network (ResNet) to alleviate the declining-accuracy effect. The 3D-ResNet model achieved very competitive classification results and increased the classification accuracy compared with the 3D-CNN model. Reference [35] also introduced the residual learning to optimize several convolutional layers. It fused the output of different hierarchical layers to improve the classification accuracy. According to dense convolutional network in References [36], Reference [37] proposed the three-dimensional densely connected convolutional network (3D-DenseNet). Through the densely connected structure, the 3D-DenseNet was deeper in structure and could learn more robust spectral-spatial features. Reference [38] adopted the capsule network composed with spectral-spatial units, which can extract spectral-spatial features through 3-D convolutions. However, the common problems existing in the 3D-ResNet and the 3D-DenseNet are that these deep learning models have complex network structure and generate huge computational parameters, which makes them hard to train. In summary, the deep learning methods based on the 3D-CNN makes full use of the structural characteristics of 3D HSI data. However, although these 3D-CNN models can extract features directly from the original HSI data, when the network becomes deeper, the degradation phenomenon will occur [39], and designing architectures becomes increasingly difficult with the growing number of hyper-parameters.

The network is usually deepened or widened to improve the accuracy of the model. However, as the number of hyperparameters increases (such as the number of channels, filter size, et al.), the difficulty and computational cost of networks will increase dramatically. Hence, Reference [40] proposed an improved structure based on the residual network, named the ResNeXt network. It can improve the accuracy by group convolutions without increasing parameter complexity and can reduce the number of hyperparameters at the same time. Meanwhile, the method, repeating a building block that aggregates a set of transformations with the same topology, simplifies the workload of the network structure design [40].

Therefore, inspired by Reference [40], we proposed an end-to-end spectral-spatial 3D ResNeXt network using feature fusion and label smoothing for HSI classification. Taking the abundant spectral features into consideration, it combines the spectral features extracted by the residual network with the HSI data after dimensionality reduction by the convolutional layer as the input of 3D-ResNeXt spectral-spatial feature learning network. Compared with spectral-spatial residual network (SSRN) network [26], our network adopts 3-D CNNs to simultaneously extract spectral and spatial features, which reduces the possibility of the information loss and improves the classification accuracy. While compared with 3D-ResNet [34], our network uses group convolution, which has smaller parameter quantity and computation cost, to extract spectral-spatial feature. Additionally, label smoothing strategy is used to modify the loss function to solve the imbalance of classes.

In short, the three major contributions of this paper are listed as follows.

1. The designed HSI classification model adopts a highly modularized network structure based on residual connections and group convolutions to mitigate the decreasing-accuracy phenomenon and reduce the number of the parameters. The whole network consists of two consecutive 3D blocks which can improve the classification accuracy of classes with relatively small number of samples.
2. Considering that HSIs have many spectral bands, the HSI data cubes after dimensionality reduction by the convolutional layer, combined with the spectral features learned by 3D-ResNet, is used as the input of the 3D-ResNeXt spectral-spatial feature learning network. This approach enriches the information of network input, especially for those classes with few samples, and is conducive to network learning more effectively.
3. Owing to the imbalance of HSI sample categories, the proposed network adopts the cross-entropy loss function modified by label smoothing strategy to improve the classification results further.

The remaining parts of this paper are organized as follows. Section 2 presents our proposed network for HSI classifications. Section 3 first illustrates experimental datasets and the network configuration, and then shows experimental results and analyses. We summarize some conclusions in Section 4.

## 2. Proposed Framework

In this section, we give a detailed description of our HSI classification network. First, we provide an overview of the proposed network architecture, and then describe the data pre-processing tactic and the structure of each block in detail. Finally, we introduce the modified loss function.

### 2.1. Overview of Proposed Network Architecture

**Figure 1:**
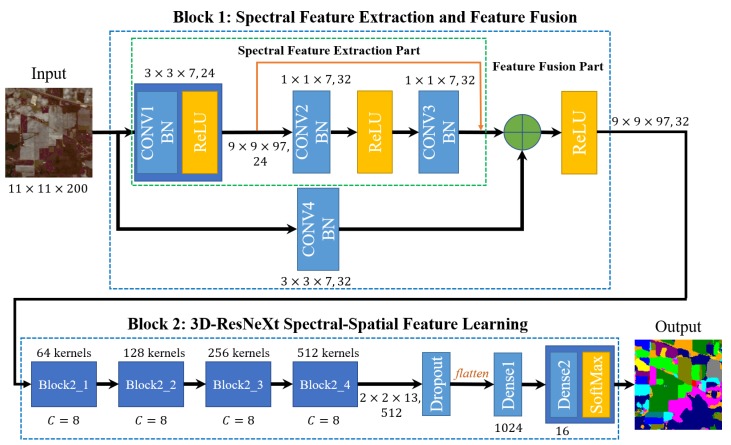
Overall structure of the proposed Hyperspectral Image (HSI) classification framework.

Figure 1 shows the network structure of 3D-ResNeXt network using feature fusion and label smoothing strategy. It consists of two major blocks: one is spectral feature extraction and feature fusion block, and the other is spectral-spatial feature learning block. The latter contains 4 same building blocks, and the structure and specific details are illustrated in Section 2.4. Block 1 is composed of spectral feature extraction part and feature fusion part. Since the classes with small number of samples have less spatial information but have many spectral bands, the former can be used to learn low-level spectral features to enhance the representation of these classes. While the latter combines the HSI data after dimensionality reduction and low-level spectral features as the input of the subsequent network. Block 2 is the main spectral-spatial feature learning network. Split-transform-merge strategy can make each transformation performing on a low-dimensional embedding and decrease the number of parameters. Moreover, we adopt a loss function modified by label smoothing to evaluate classification performance. To take advantage of both spectral and spatial information, we feed the network with a window containing a small neighborhood of pixels. In Figure 1, we take Indian Pines (IN) dataset with 11×11 patch as the input to illustrate the size of feature maps used in our network.

### 2.2. Data Preprocessing

**Figure 2:**
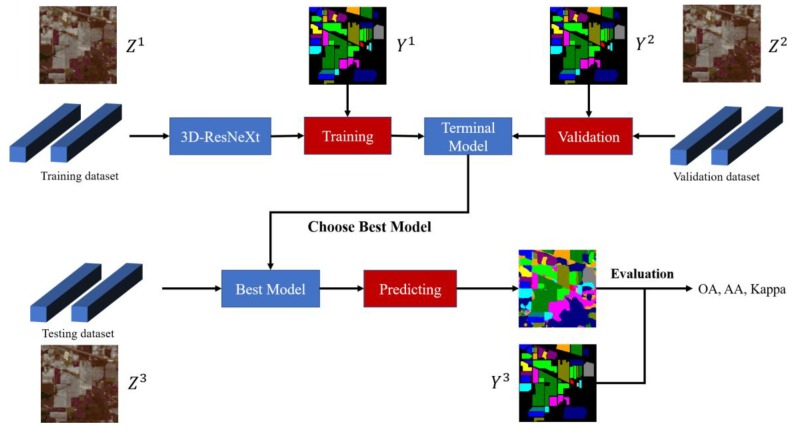
The end-to-end HSI classification flowchart.

The proposed end-to-end deep learning flowchart of HSI classification is illustrated in Figure 2. Note that all available labeled data for different object classes are divided into three groups: training dataset $Z^{1}$, validation dataset $Z^{2}$, and testing dataset $Z^{3}$, and the corresponding one-hot label vectors are $Y^{1}$, $Y^{2}$, and $Y^{3}$, respectively. The desired label for the network is the class of the centered pixel. First, the ground-truth labels are shuffled and divided into training set label ($Y^{1}$) and whole testing set label Y ($Y^{2}$ and $Y^{3}$) in proportion (i.e., the percentage of the sum of testing and validation sets). Note that at this time test validation sets have not been separated. And then according to these two sets, the raw HSI data is processed and divided into corresponding training dataset $Z^{1}$ and testing dataset Z ($Z^{2}$ and $Z^{3}$). To fully utilize the spatial information from HSI data, especially at the border, zero-padding strategy is used to extend the boundaries of the original image X to form a new group of the dataset $Z\in R^{w\times w\times b}$, where w represents the spatial sizes and b represents the number of spectral bands. For example, IN dataset has 145×145 pixels in spatial domain, with 200 available spectral bands. Due to the input size of HSI cubes for IN classification is 11×11×200, 5 pixels are respectively expanded in four directions of the two-dimensional space, and then the size has changed to 155×155×200. Through boundary expansion, the network can learn the entire dataset even the boundary information. Finally, validation dataset $Z^{2}$ and its corresponding one-hot label vector $Y^{2}$ are separated from the whole testing sets Y and Z according to the proportion.

After determining the architecture and parameters of the model, by feeding the training group $Z^{1}$ and its corresponding ground-truth label vector set $Y^{1}$, the models are trained for hundreds of epochs. Meanwhile, the validation set $Z^{2}$ monitors the training process by testing the classification accuracy and the loss of the temporary model. Early stopping method is employed to prevent overfitting. During this process, the network with the highest classification accuracy is selected to retain the corresponding weight parameters of models. Finally, the testing group $Z^{3}$ is used to evaluate the prediction performance by calculating overall accuracies (OA), average accuracies (AA), and kappa coefficients (Kappa) between model prediction and ground-truth labels $Y^{3}$.

### 2.3. Block 1: Spectral Feature Extraction and Feature Fusion

In Block 1, considering that HSI data has much important spectral information, we first use 3D-ResNet to learn spectral feature, followed by the feature fusion part. At the beginning of the spectral feature extraction part and feature fusion part, we both use the 3-D convolutional layers with the size of 3×3×7 to appropriately reduce the dimensionality of the original HSI data. 

**Figure 3:**
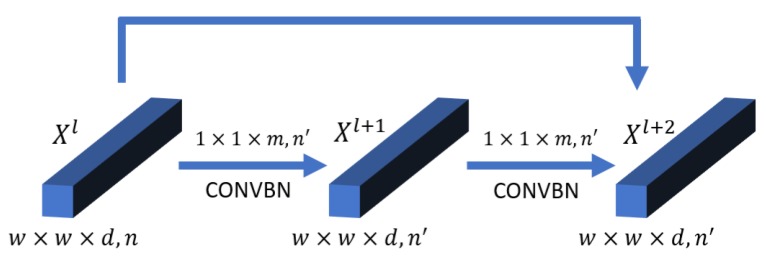
Three-dimensional spectral residual block to extract spectral features.

The spectral feature extraction part includes a convolutional layer and a spectral residual block. Firstly, 24 3×3×7 spectral-spatial kernels with a subsampling stride of (1, 1, 2) convolves the raw HSI data to generate 24 9×9×97 feature cubes. This layer reduces the high dimensionality of HSI data and extracts low-level spectral-spatial features of raw HSI data. Then in the spectral residual block, which is shown in Figure 3, we set the size of 3-D convolutional kernels 1×1×m (in our experiment m=7) to only extract the feature of spectrum dimension and keep the spatial size of output feature cubes the same as input. Finally, we can get 32 9×9×97 spectral feature maps as the output of the spectral feature extraction part.

While in the feature fusion part, the convolutional layer reduces the dimensionality of the raw HSI data with 32 3×3×7 spectral-spatial kernels with a subsampling stride of (1, 1, 2). This layer ensures that the size of output feature cubes is the same as the output size of the spectral feature extraction part. Then an Add layer can easily merge the 3D data after dimensionality reduction and the spectral features learned by 3D-ResNet. Therefore, final 32 9×9×97 feature maps containing much spectral features and low-level spectral-spatial features are performed as the input of the subsequent 3D-ResNeXt spectral-spatial feature learning part.

In summary, this block can reduce the degradation phenomenon of classes imbalance effectively. If we only use the HSI data processed by dimensionality reduction as the input of the network, it may result in the information loss mainly coming from classes with few samples. And as the depth of the network increases, the feature maps of classes with small number of samples will become fewer and fewer, so that the network cannot effectively learn these features. Finally, it may lead to the low classification accuracy of classes with few samples. However, our proposed block could solve this problem by enriching the input of the network. Owing to the rich spectral bands of HSI data, even for the few land-cover categories, we can combine the HSI data after dimensionality reduction by the convolutional layer and the spectral feature extracted from the residual network (ResNet). Then the data cubes after the feature fusion are used as the subsequent input of the ResNeXt spectral-spatial feature learning network. This way can enrich the input of network, especially the spectral features of few land-cover classes, and can ensure that each category even with few samples can be classified efficiently and correctly.

### 2.4. Block 2: 3D-ResNeXt Spectral-Spatial Feature Learning

**Figure 4:**
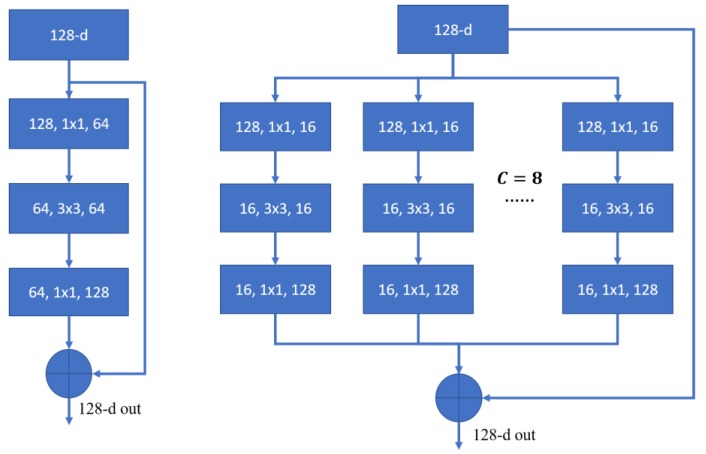
A block of ResNet (Left) and ResNeXt with cardinality = 8 (Right). A layer is shown as (# in channels, filter size, # out channels).

Considering that HSI datasets are three-dimensional cubes, 3D-CNN is taken into our network to learn spectral-spatial features. The proposed 3D-ResNeXt spectral-spatial feature learning network is constructed by repeating a building block that has the same topology. As shown in Figure 4, the left one is a block of ResNet and the right one is a block of ResNeXt with cardinality = 8, with roughly the same complexity [40]. We can find that the split-transform-merge strategy makes each transformation performing on a low-dimensional embedding, whose outputs are aggregated by summation [40]. It can reduce the number of parameters of each 3-D convolution filters and extract more spectral-spatial features without very deep network.

**Figure 5:**
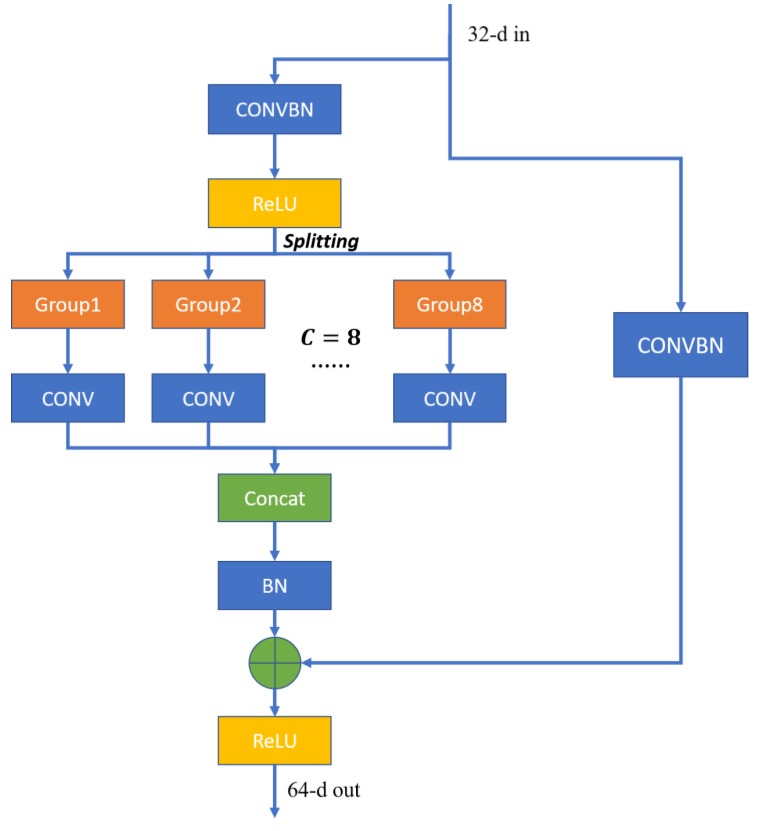
General structure of a ResNeXt block with cardinality = 8 (Taking the Block2_1 for example).

In the experiment, we adopt four same building blocks to construct a deep network. With reference to the design scheme of Reference [40] and the experiment of cardinality in Section 3.3.2, we set the hyperparameter cardinality *C* = 8 and the total numbers of filters of four building blocks to {64, 128, 256, 512} respectively. Therefore, the numbers of filters in each block which are used to learn the spectral-spatial features are {8, 16, 32, 64}, respectively. Meanwhile, we set stride = 2 in the last three blocks to reduce the spatial dimensionality. Figure 5 shows the structure of one sub-block and the others have the similar block structure. First, a convolution layer with the size of 1×1×1 is used to change the dimensionality of the feature channel. Then, through splitting operation, input data are transformed to some small blocks, which are transferred to the convolution layers to learn the features. Finally, a concatenate layer aggregates the processed data into a high-dimensional feature vector again. While in the shortcut part, in order to make sure that the feature vectors of two parts have the same size, the convolution layer is used to change the dimensionality of input data.

We perform batch normalization (BN) [41] right after the convolutions to prevent the gradient dispersion problem. ReLU [42] is performed after the adding to the shortcut. In order to further address the overfitting problem, a dropout operation, which sets the output maps generated by some neurons to zeros with a specific probability [43], is used at the end. In this work, the dropout rate is set to 0.5, which is commonly used in deep learning models. Finally, after the full-connected layers, we use a SoftMax layer to obtain the classification results.

There are two main reasons which encourage us to use the ResNeXt structure instead of the ResNet structure for HSI classification tasks. First, the ResNet network might bring large number of parameters when using 3D convolution layers because of more than 100 spectral bands of HSI data. It may make the network difficult to train. However, our proposed network splits the channels to many small blocks, and then each block extracts spectral-spatial features by 3-D convolution. Group convolutions in HSI spectral-spatial feature extraction reduce the number of parameters and computations to a large extent. Therefore, to extract more representative spectral-spatial features, we can increase the number of groups (i.e., the hyperparameter cardinality *C*) instead of the depth of network. The specific comparison experiment details can be found in Section 3.4. Second, as the number of 3-D convolution filters in each group increases because of the hyperparameter C, the input of each convolution is different, which can increase the locality of different features. More convolution operations can extract more representative spectral-spatial features. In other words, compared with the ordinary convolutions which can only generate one feature map, C feature maps can be generated by group convolutions with the same number of parameters and operations. More feature maps mean more spectral-spatial features extracted by the network and more efficiently learning features, which may result in the higher classification accuracy finally. In summary, the ResNeXt network based on split-transform-merge strategy and group convolution has been demonstrated to learn better representations of the HSI data to achieve better results than the 3D-ResNet network.

### 2.5. Modified Loss Function

In the image classification tasks, the last layer of network usually is a SoftMax layer. Denoted by the predicted score for class i, $z_{i}$ can be normalized by the SoftMax operator to obtain predicted probabilities [44]. The ultimate probability for class i, $q_{i}$ can be computed by:

(1) $$q_{i}=\frac{exp\left(z_{i}\right)}{\sum_{j=1}^{K}exp\left(z_{j}\right)},$$

where K is the number of classes. It is easy to see that $q_{i}$ ranges from 0 to 1, and

(2) $$\sum_{j=1}^{K}q_{j}=1,$$

In general, the truth probability distribution $p_{i}$ satisfies

(3) $$p_{i}=\{\begin{array}{c} 1,\;\;\;if\;\;i=y\\ \;\;0,\;otherwise \end{array},$$

Then we can use cross entropy loss function to minimize the negative cross entropy loss by gradient descent. The formula is as follows:

(4) $$L=-\sum_{i=1}^{K}p_{i}logq_{i}=-logq_{y},$$

We can find that the optimal solution of (4) is $z_{y}^{*}=inf$ while keeping others small enough [45]. This may bring two problems: first, since that the data used for training is usually limited, for example in HSI classification tasks, the number of Indian dataset that have been labeled is only around 10,000, which means that the number of the training dataset is usually less than 5,000, and the problem of the imbalance of HSI dataset exists, the prediction of the model may deviate from the real situation, which may lead to the low accuracy. Second, one-hot encoding encourages the differences between the largest logit, and it may result in overfitting because the model becomes too confident about its predictions [46].

Therefore, Reference [45] proposed a modified cross entropy loss function. It used soft targets that are a weighted average of the hard targets and the uniform distribution over labels [46]. Then the truth probability distribution becomes

(5) $$p_{i}^{'}=\left(1-\varepsilon \right)p_{i}+\varepsilon u\left(K\right)=\{\begin{array}{c} 1-\varepsilon +\varepsilon /K,if\;i=y\\ \varepsilon /K,\;otherwise \end{array},$$

where ε is a small constant, and in our task, for the convenience of experiments, we set ε 0.1. Now the loss function becomes

(6) $$L=-\sum_{i=1}^{K}p_{i}^{'}logq_{i}=-(1-\varepsilon +\varepsilon /K)logq_{y}-\frac{\varepsilon }{K}\sum_{i\neq y}logq_{i},$$

We can see that the loss function contains the $q_{i}$ of the incorrect class, and according to Reference [45], the optimal solution is not $z_{y}^{*}=inf$ anymore. This encourages a finite output from the fully-connected layer [44].

In the HSI classification task, the HSI datasets usually show the phenomenon of class imbalance, which may lead to the low classification accuracy of classes with few training samples. Label smoothing regularization, to a certain degree, can prevent the model from ignoring the classes with low probability distribution. The method that adds an extra priori probability as the penalty can balance the probability distribution between the classes with large number and small number of samples. Due to the rich spectral features of HSI datasets, we can use label smoothing strategy to improve the probability distribution of classes with few samples, and the model can learn the spectral-spatial features of these classes more effectively.

As far as we know, label smoothing strategy has not been used in previous HSI classification methods based on the deep learning. In our experiment, we compared the results obtained by the modified loss function with those obtained by the ordinary loss function. From the results, we find that this tactic can indeed improve the average accuracies of classes which have very few samples for our HSI classification task. The specific implementation details and results are illustrated in Section 3.4.

## 3. Experiments and Results

In this section, we first illustrate three HSI datasets and configurations of the experiments. Then we discuss the impact of different network parameters. To evaluate the HSI classification performance of the proposed model, we compare the proposed model with recent representative HSI classification models which have been introduced in Section 1, such as SVM [12], rank-1 FNN [14], 3D-CNN [21], SSRN [26], and 3D-ResNet [34].

### 3.1. Experimental Datasets

Three publicly available datasets [47] are used in the experiment to evaluate the performance of the proposed model. Table 1, Table 2 and Table 3 show the total number of samples of each class in each dataset

The Indian Pines (IN) dataset [48], collected by the Airborne Visible/Infrared Imaging Spectrometer (AVIRIS) in 1992 from Northwest Indiana, includes 16 vegetation classes. Its size is 145×145 pixels with a spatial resolution of 20 m by pixel. There are 220 bands in the wavelength range of 0.4 to 2.5 um. But 20 bands corrupted by water absorption effects were discarded, the remaining 200 bands are used for experiments.

The Pavia University (UP) dataset [48] was gathered by Reflective Optics System Imaging Spectrometer (ROSIS) in 2001 in the Pavia region of northern Italy. It has 610×340 pixels with a resolution of 1.3 m by pixel and contains 9 types of geographic objects. Once removing 12 bands containing strong noise and water vapor absorption, the other 103 bands ranging from 0.43 to 0.86 um are employed for training and evaluation.

The Kennedy Space Center (KSC) [49], acquired by AVIRIS in 1996 in the Kennedy Space Center, contains 224 bands with center wavelengths from 0.4 to 2.5 um. It has 512×614 pixels with a spatial resolution of 18 m. After removing water absorption and low signal-to-noise ratio (SNR) bands, 176 bands are used for the analysis. There are 13 classes representing the various land cover types.

### 3.2. Experimental Setup

We tested the factors that affect the HSI classification performance of the network, and the specific experimental details were illustrated in Section 3.3. In each block, we investigated the optimal number of filters and composite functions. Finally, the most suitable spatial size was 11×11, and the cardinality (the size of the set of transformations) was 8. Taking IN dataset as an example, the detailed network parameter setting for three HSI datasets is shown in Table 4. We adopted the RMSProp as the optimizer to minimize the modified cross-entropy loss function. The initial learning rate was set to 0.0003. All the training and testing results were obtained on the same computer, with the configuration of 16GB of memory, NVIDIA GeForce GTX 1070 8GB and Intel i7 7820HK. 

We chose the overall accuracy (OA), average accuracy (AA), and kappa coefficient (Kappa) as the standards of measuring network performance. At the same time, to demonstrate the classification effectiveness of the network for the small number of training samples, we showed the Precision, Recall, and F1-Socre indicators of classes with the smallest number of samples in the three HSI datasets (i.e., Class 9 Oats for IN dataset, Class 9 Shadows for UP dataset, and Class7 Swamp for KSC dataset). To obtain a statistical evaluation, each experiment was repeated 5 times, and we calculated the mean value as the final results.

### 3.3. Experimental Parameter Discussion

We focus on the three factors that control the training process and influence the classification results, including the ratio of the training dataset, the input spatial size, and the cardinality. Finally, for the IN, UP, and KSC dataset, the ratios of training, validation and test datasets are {5:1:4, 4:1:5, 5:1:4}, respectively. At the same time, the input spatial size and the cardinality are also optimal, which are 11×11 and 8 respectively.

#### 3.3.1. Effect of Different Ratios of Training, Validation and Test Datasets

**Figure 6:**
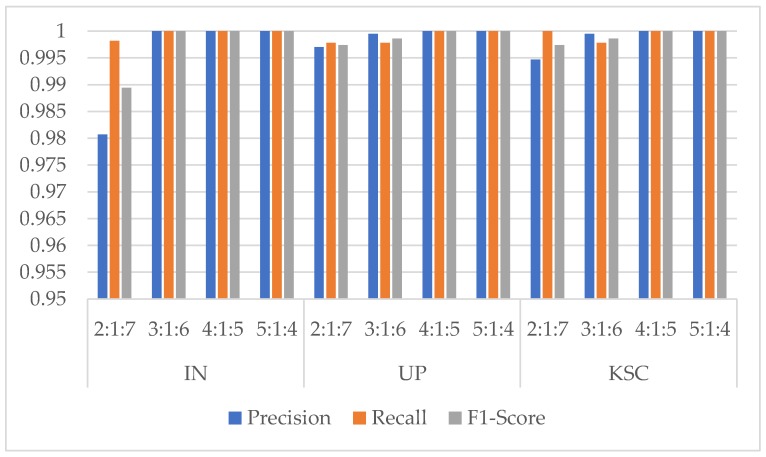
Precision, Recall, and F1-Score indicators of classes with the smallest number of samples under different training ratios in the three HSI datasets. (Class 9 Oats for IN dataset, Class 9 Shadows for UP dataset, and Class 7 Swamp for KSC dataset).

In order to test the impact of the number of training samples on models, just like the experiment of the effect with different ratios of training samples in Reference [37], we also divided the datasets into different proportions (2:1:7, 3:1:6, 4:1:5, 5:1:4). To obtain the best results, the epochs of different ratios are {100, 80, 60, 60}, respectively. At this time, the spatial size was fixed at 11×11, and the cardinality was 8. The training time, test time, and overall accuracy (OA) changes are shown in Table 5, Table 6 and Table 7 for different ratios by the proposed model. And Figure 6 shows the Precision, Recall, and F1-Socre indicators of the categories with the smallest number of samples under different ratios in the three HSI datasets.

From Table 5, Table 6 and Table 7, we can see that with the increasing of the training samples, three HSI datasets all showed a clear increasing trend in OA indictors. For IN and KSC datasets, when the ratio was 5:1:4, the OA indictor was the highest, and the training time did not increase much compared to those with other ratios. In addition, owing that the IN and KSC datasets are small, we used a larger training dataset to improve the accuracy and the training process is more stable. Therefore, we finally chose the ratio of 5:1:4 for IN and KSC datasets in our proposed network. However, for the UP dataset, with the increasing number of training samples, the training time rose rapidly, whereas the accuracy rose little. Especially when the ratio changed from 4:1:5 to 5:1:4, the training time showed a dramatic jump from 6857.42s to 8630.59s, whereas the overall accuracy kept almost unchanged at 99.99%. It means that we may consume more computing time and resources to get less classification performance improvements. On the other hand, the UP dataset has more than 4,000 samples, so we do not need too many training samples to train the model. Finally, we chose the ratio of 4:1:5 for the UP dataset. What’s more, we may notice that when the training samples reached 30%, the OA indictors of the proposed network in the three HSI datasets were all higher than 99.80%, which had nearly reached the highest classification accuracy compared with the previous methods. However, we still chose the higher ratio of training samples because the proposed network is deeper than others, so more samples are used trained for the network, more features could be extracted by group convolutions. The OAs prove the superiority of our method.

From Figure 6, we can find that the three indicators are all higher than 0.95 in three HSI datasets. In the IN dataset, when there are few training samples for these classes, the indicators are slightly lower. However, when the ratio comes to 5:1:4, the Precision, Recall, and F1-Socre indicators of these classes are nearly close to 1. It means that the proposed model with the ratios of 5:1:4 for IN dataset shows a good classification performance. While in UP and KSC datasets, the phenomenon of class imbalance is not obvious, so the Precision, Recall, and F1-Socre indicators are all close to 1. In summary, the best choice for the ratios of three HSI datasets are {5:1:4, 4:1:5, 5:1:4} respectively, and the proposed model could improve the classification performance of classes with few samples.

In addition, we may notice that the training time of the ratio of 3:1:6 is higher than that of 4:1:5. The reason is that considering that when the ratio is 3:1:6, the number of the training samples is small. Therefore, we set the ratio of 3:1:6 to 100 epochs to obtain the best results. While when the ratio becomes 4:1:5, we do not need too many epochs to train the model because we use relative more training samples in each epoch.3.3.2. Effect of Input Spatial Size

In general, with the increase of the input spatial size of network input, the number of parameters also increases. If the input spatial size is too small, the receptive field is likely to be too insufficient to achieve a good result. Therefore, in the experiment, we selected five different spatial sizes ranging from 7 to 15 to evaluate the influence of the size of network input and choose the best one as the hyperparameter for the comparative experiments. In order to make full use of all the spatial data, the corresponding boundary padding spatial sizes are {3, 4, 5, 6, 7}. Taking IN dataset as an example, the network fills the boundary of the original input 145×145×200 image with padding spatial size 5 into 155×155×200 images.

**Figure 7:**
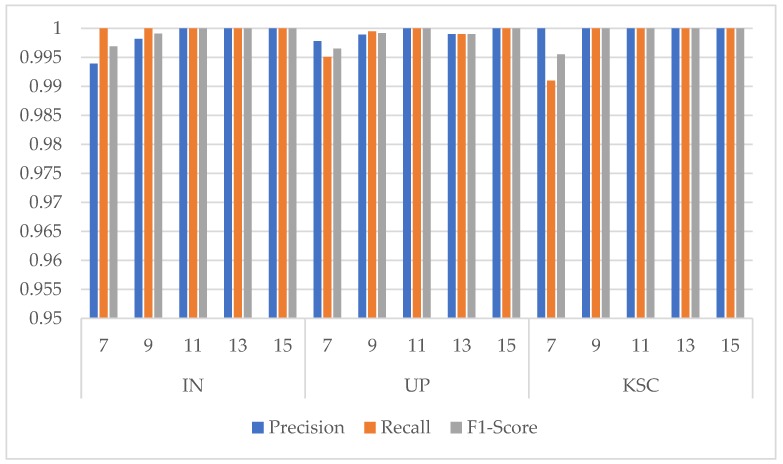
Precision, Recall, and F1-Score indicators of classes with the smallest number of samples under different input spatial size in the three HSI datasets. (Class 9 Oats for IN dataset, Class 9 Shadows for UP dataset, and Class 7 Swamp for KSC dataset).

As shown in Table 8, Table 9 and Table 10, for the IN dataset, the overall accuracy showed a trend of first increase and then decrease slightly. While for the UP and KSC datasets, as the size of pixel blocks increased, the OA grew by a small margin. However, the training time and test time both increased dramatically, which led to the computation cost showing a huge jump. Finally, 11×11 was selected as the input spatial size on the three datasets. Moreover, from Figure 7 we can find that the three indicators for the three HSI datasets are all higher than 0.99. It means that the classification performance of small samples is not very sensitive to the input spatial size.

#### 3.3.2. Effect of Cardinality

For the proposed 3D-ResNeXt HSI classification model, the group convolution is the most important part, and the cardinality (*C*, i.e., the size of the set of transformations) is a major factor that affects network training. Reference [40] shows that even under the restricted condition of maintaining complexity, increasing cardinality is still able to improve classification accuracy. If *C* is chosen too high, owing to the total number of filters is fixed, the number of sub-filters will be too small for the network to learn the features efficiently. On the other hand, if *C* is chosen too small, it will lead to the increasing of the number of parameters and the computing power.

We evaluated the performance of the network with different cardinality and the results are listed in Table 11. In this experiment, we set the ratio 5:1:4 for the IN and KSC datasets and 4:1:5 for the UP dataset, and the input spatial size was set 11×11. To enable that the total filters of block 2 can be evenly divided into C groups, which means that the total filters can be divided by *C*, we set different numbers of filters in the block 2 for different cardinalities: {48, 96, 192, 384} for C=6, {64, 128, 256, 512} for C=8, and {80, 160, 320, 640} for C=10.

**Figure 8:**
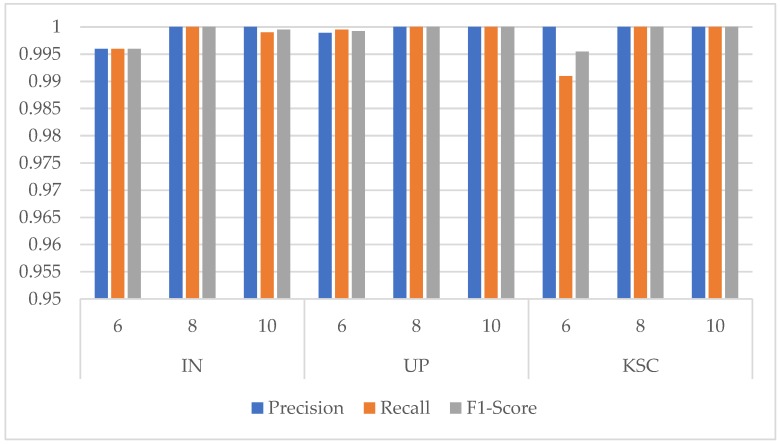
Precision, Recall, and F1-Score indicators of classes with the smallest number of samples under different cardinality in the three HSI datasets. (Class 9 Oats for IN dataset, Class 9 Shadows for UP dataset, and Class 7 Swamp for KSC dataset).

The experiment found that the cardinality has an obvious influence on the OA, showing a wave-like trend, whereas the parameter quantity, training time, and test time all rocketed. While from Figure 8 the Precision, Recall, and F1-Score indicators are all close to 1 for the classes with few samples in the three HSI datasets. It can be seen that the proposed network could stably classify the classes with small number of samples. Integrating the influence of various factors, we finally chose the cardinality of 8 for the three datasets.

### 3.4. Classification Results Comparison with State-of-the-Art

Due to the rich spectrum information of HSIs, spectral feature-based HSI classification is a traditional and effective method, in which 1D-CNN focusing on spectral domain is widely used. On the other hand, the original CNN is designed for 2-D image classification. The performance of 2D-CNN for HSI classification should be tested and compared. In summary, 1D-CNN, 2D-CNN-LR, 3D-CNN are all adopted previously for spectral, spatial, and spectral-spatial feature based HSI classification, respectively. 2D-CNN-LR adopted the logistic regression (LR) as the single-layer classifier. Therefore, in our experiments, we compared our proposed 3D-ResNeXt model with classic SVM [12], rank-1 FNN [14], 1D-CNN [21], 2D-CNN-LR [21], 3D-CNN [21], SSRN [26], and 3D-ResNet [34] model. To make a fair comparison, we set the same input spatial size of 11×11×b (b represents the number of spectral bands), the ratio 5:1:4 for the IN and KSC datasets and 4:1:5 for the UP dataset for all methods. As seen from the experiment effects with different ratios of training samples in Section 3.3, when the ratio comes to 3:1:6, the overall accuracies of the three HSI datasets by our method have exceeded 99.80%, which has generated better classification performance than other methods. To make a fair comparison and demonstrate the superiority of our proposed network further, we set the same input spatial size of 11×11×b, the ratio 5:1:4 for the IN and KSC datasets and 4:1:5 for the UP dataset for all methods instead of 3:1:6.

First, we compared the proposed network with SVM, rank-1 FNN, 1D-CNN, 2D-CNN-LR, 3D-CNN, SSRN, and 3D-ResNet. From Table 12 we can see that the traditional machine learning based method SVM got the worst classification accuracy, and it means that the traditional methods using feature extraction by manual often get relatively poor performance in HSI classification tasks. On the other hand, rank-1 FNN could achieve higher classification accuracies than 1D-CNN and 2D-CNN-LR. It demonstrated that rank-1 FNN could learn spectral and spatial features better by utilizing a linear and a nonlinear tensor-based scheme. What’s more, the networks based on 3-D CNN obtain significantly better classification performance. Therefore, we further compared the performance of these networks in detail.

**Figure 9:**
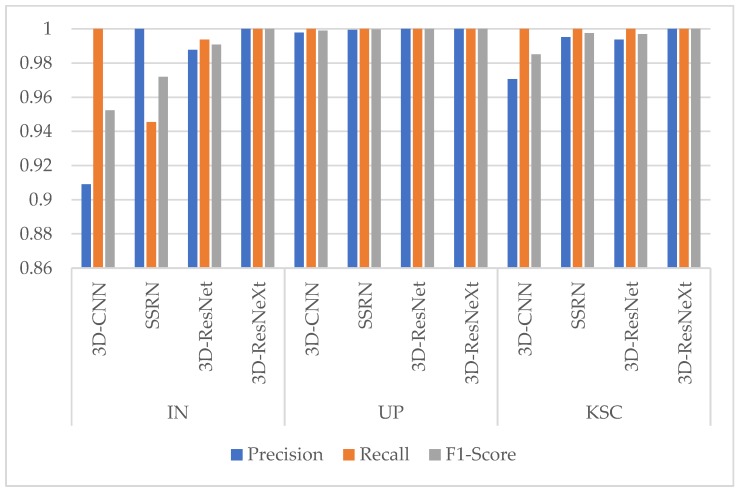
Precision, Recall, and F1-Score indicators of classes with the smallest number of samples of four models using 3-D Convolutional Neural Network (CNN) in the three HSI datasets. (Class 9 Oats for IN dataset, Class 9 Shadows for UP dataset, and Class 7 Swamp for KSC dataset).

Table 13, Table 14 and Table 15 report the OAs, AAs, kappa coefficients, and the classification accuracies of all classes by different methods based on 3-D CNN for three HSI datasets. From Table 13, Table 14 and Table 15, we can see that, the 3D-ResNeXt achieved the highest classification accuracy than other methods for all three datasets. For example, in the IN dataset, 3D-ResNeXt achieved a roughly 0.8% increase of OA, AA, and kappa coefficient compared with SSRN. Compared with the 3D-ResNet, which had achieved relatively high accuracies, our proposed network achieved better indicators based on the 3D-ResNet results. The classification accuracies of the networks that only focus on spectral or spatial features reached less than 95% usually. However, all the methods based on 3D-CNN generated obviously better outcomes than traditional methods and the networks that only focus on one dimensionality (spectrum or space) information. It indicated that ignoring the information of any dimension cannot achieve the desired result. In the IN and KSC datasets, 3D-CNN got better result than SSRN, and the classification accuracies of SSRN on classes 1, 7, and 9 (Alfalfa, Grass-pasture-mowed, and Oats, respectively) are lower than 3D-CNN. The training samples of these classes are extremely small, and SSRN that has spectral and spatial feature learning parts is not reliable for the identification of small HSI samples. Furthermore, although the classes 1, 7, and 9 (Alfalfa, Grass-pasture-mowed, and Oats, respectively) have few training samples, the 3D-ResNeXt could still classify these classes with higher than 98% classification accuracies. It means that the designed model has a good robustness and can still achieve good performance in the face of small samples. And Figure 9 shows the Precision, Recall, and F1-Socre indicators of these four models using 3-D CNN to extract spectral and spatial features under the same parameter setting. It can be seen that in the three HSI datasets the indicators of 3D-CNN model are the worst of these four models, followed by SSRN. Especially in the IN dataset, these two models could not achieve good classification performance for classes with small samples. However, our proposed model could accurately classify each class with indicators close to 1 in all three HSI datasets.

**Figure 10:**
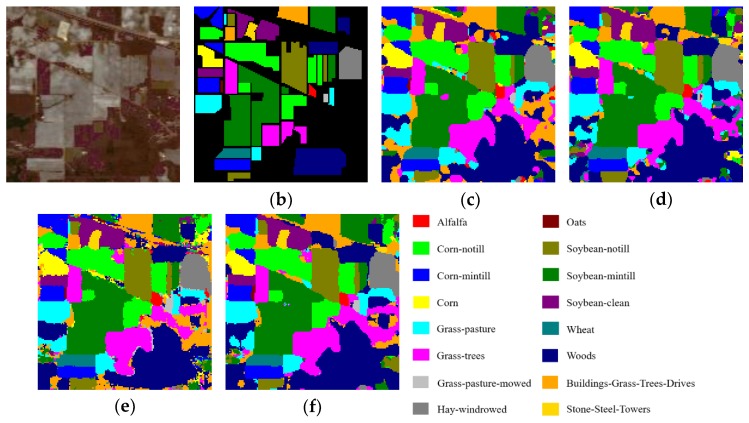
Classification results of the models in comparison for the IN dataset. (**a**) False color image, (**b**) Ground-truth labels, (**c**)–(**f**) Classification results of 3D-CNN, SSRN, 3D-ResNet, and 3D-ResNeXt.

**Figure 11:**
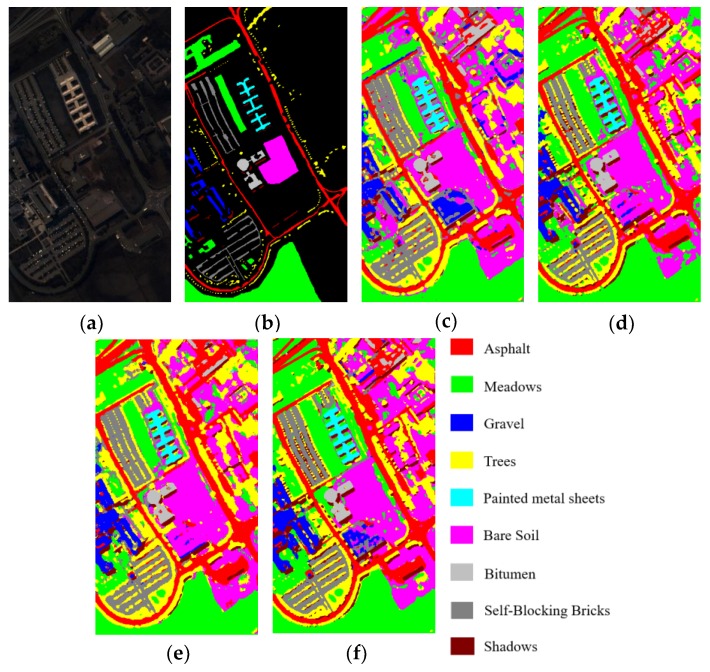
Classification results of the models in comparison for the UP dataset. (**a**) False color image, (**b**) Ground-truth labels, (**c**)–(**f**) Classification results of 3D-CNN, SSRN, 3D-ResNet, and 3D-ResNeXt.

**Figure 12:**
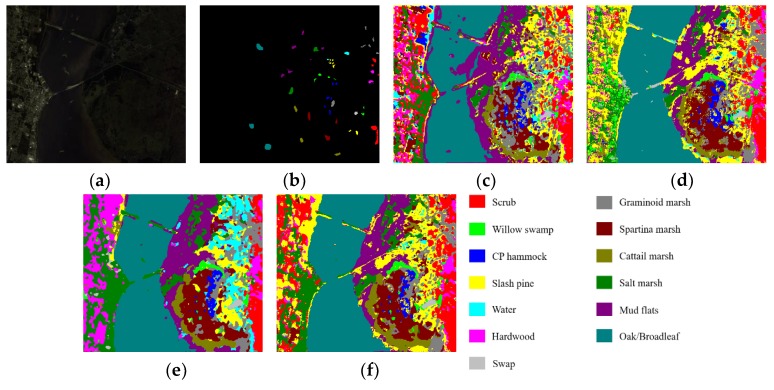
Classification results of the models in comparison for the KSC dataset. (**a**) False color image, (**b**) Ground-truth labels, (**c**)–(**f**) Classification results of 3D-CNN, SSRN, 3D-ResNet, and 3D-ResNeXt.

Figure 10, Figure 11 and Figure 12 visualize the classification maps of the best four methods (3D-CNN, SSRN, 3D-ResNet, and 3D-ResNeXt) in comparison, along with the false color images of original HSI and their corresponding ground-truth maps. In the three HSI datasets, 3D-CNN had a partial misjudgment for unmarked samples. For example, we can find that for the UP dataset the part marked in blue in the middle of Figure 11 (c) is actually Class 8 that is not labeled, while 3D-CNN incorrectly labeled it as Class 3. 3D-ResNet generated classification maps with strong noise, especially in the IN and KSC datasets. SSRN and 3D-ResNeXt could effectively remove these misclassification noises. However, SSRN reduced the color maps in the classes 7 and 9 (Oats class and Grass-pasture-mowed, respectively) of the IN dataset. Compared with the other three methods which had high classification accuracies, the 3D-ResNeXt delivered the most accurate classification maps, and the edge contours of geographic objects are clearer than others. For example, for the IN dataset, Class 1 and 9 have the smallest number of samples, but our proposed network can still completely classify these two classes. Specifically, comparing the ground-truth labels and the classification figure (Figure 10f), we can see that both red and dark red parts are completely displayed.

Regarding the computational cost of different HSI classification networks, training time and test time are listed in Table 16, Table 17 and Table 18. It can be clearly seen that SSRN required the least time for training and test, because there are few convolutional layers in SSRN to learn spectral and spatial features. For the networks based on 3-D convolution, 3D-CNN consumed less time than 3D-ResNet and 3D-ResNeXt. However, it could not achieve high classification performance compared with 3D-ResNet and 3D-ResNeXt. Since the proposed network is deep, it takes longer training time and test time to learn spectral-spatial features than 3D-ResNet. However, the proposed network obtained the highest classification accuracies for the three HSI datasets.

**Figure 13:**
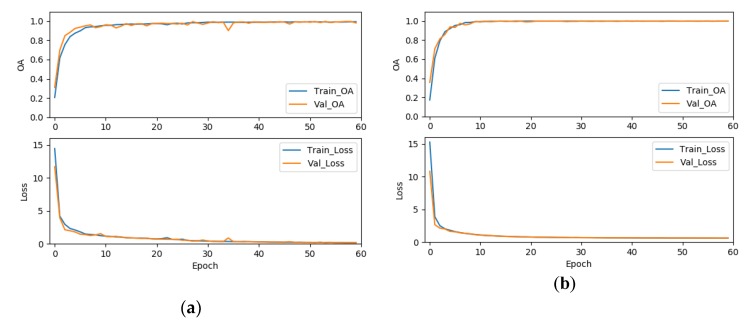
The OA and loss of models with different loss functions for the IN dataset, (**a**) the original cross-entropy loss function, (**b**) the cross-entropy loss function modified by label smoothing strategy.

**Figure 14:**
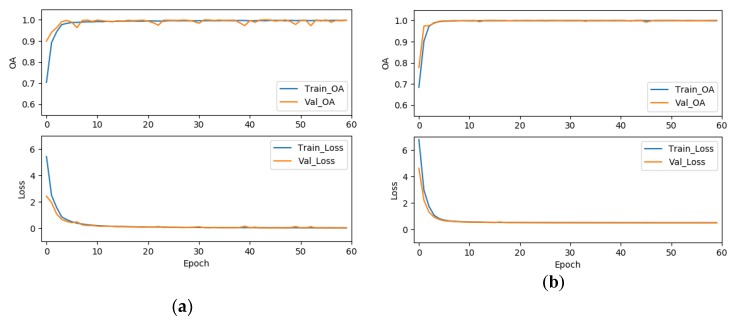
The OA and loss of models with different loss functions for the UP dataset, (**a**) the original cross-entropy loss function, (**b**) the cross-entropy loss function modified by label smoothing strategy.

**Figure 15:**
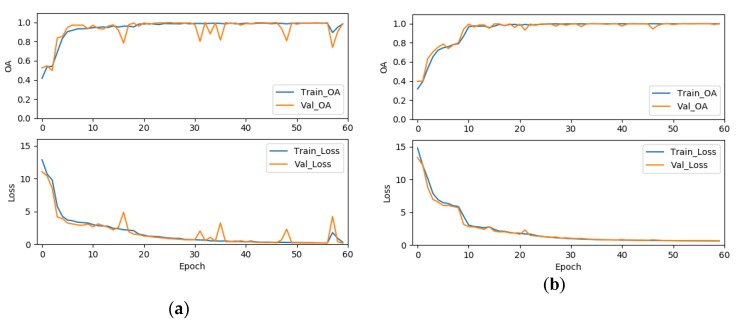
The OA and loss of models with different loss functions for the KSC dataset, (**a**) the original cross-entropy loss function, (**b**) the cross-entropy loss function modified by label smoothing strategy.

**Figure 16:**
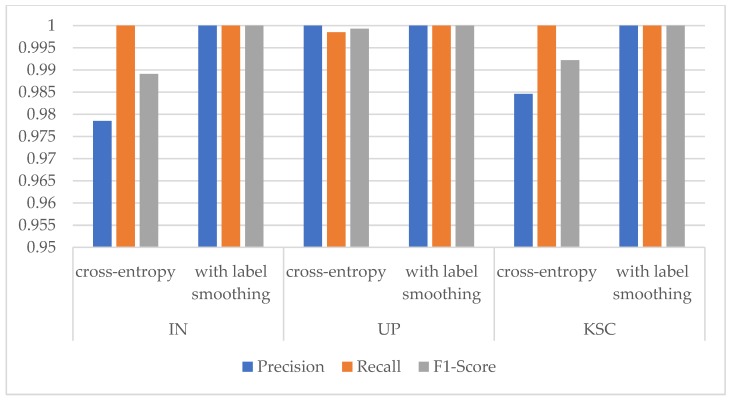
Precision, Recall, and F1-Score indicators of classes with the smallest number of samples with different loss functions in the three HSI datasets. (Class 9 Oats for IN dataset, Class 9 Shadows for UP dataset, and Class 7 Swamp for KSC dataset).

To validate the effectiveness of the label smoothing strategy for improving the classification accuracy, we further compared the results of the 3D-ResNeXt using label smoothing strategy with the network with same structure but using the original cross-entropy loss function. To achieve the convincing results, two experiments were set to have the same epochs, ratio of training dataset, cardinality, and the same input spatial size. As shown in Table 19, we could clearly find that the OA, AA, and kappa coefficient of our proposed network using the modified loss function were all about 1% higher than that without the modified loss function. And the average accuracies also demonstrated an improvement for all the classes with the network using the modified loss function by label smoothing. In addition, Figure 13, Figure 14 and Figure 15 shows the overall accuracy and loss curves of models with different loss functions. The OA and loss curves of the original cross-entropy loss function fluctuate more obviously in the IN and UP datasets. For the KSC dataset, although at the beginning the accuracy and loss of the loss function modified by label smoothing fluctuate up and down a little more obviously compared with that of the original cross-entropy loss function. However, as the epoch increases, the curves tend to be stable and the accuracy of with label smoothing strategy is higher than that of the original loss function. It means that the model using label smoothing strategy is more adaptable to HSI datasets which usually show the phenomenon of class imbalance. And Figure 16 shows the Precision, Recall, and F1-Socre indicators for the classes with smallest number of samples in the three HSI datasets. We can find that the classification performance for small samples of the network with label smoothing strategy is better than that with the original cross-entropy loss function. In summary, the label smoothing regularization is of benefit to improve the classification accuracy of classes with relatively small numbers of samples.

**Figure 17:**
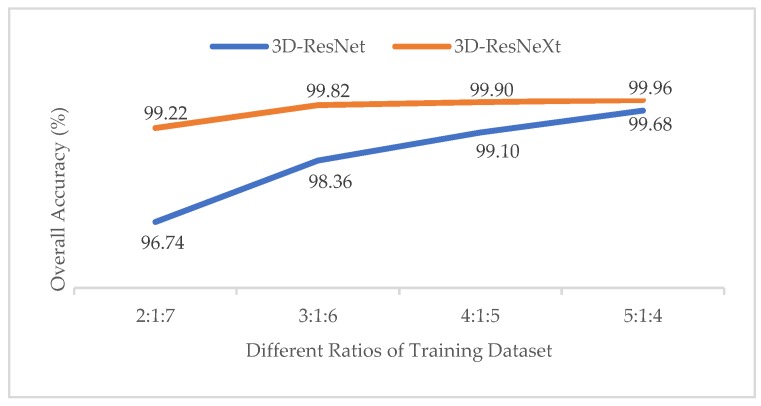
OAs of the 3D-ResNet and 3D-ResNeXt with different ratios of training samples for the IN dataset.

**Figure 18:**
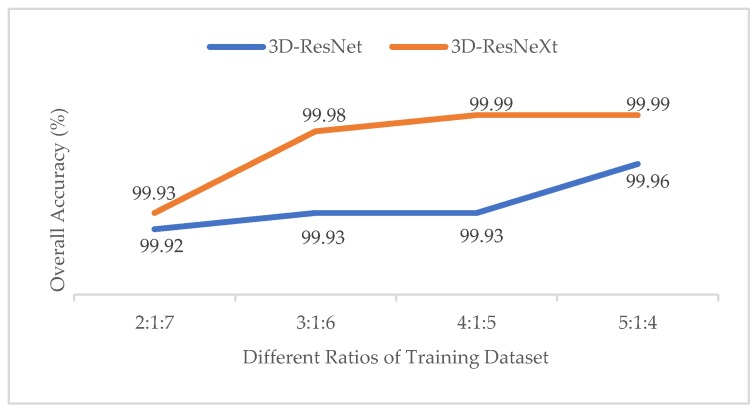
OAs of the 3D-ResNet and 3D-ResNeXt with different ratios of training samples for the UP dataset.

**Figure 19:**
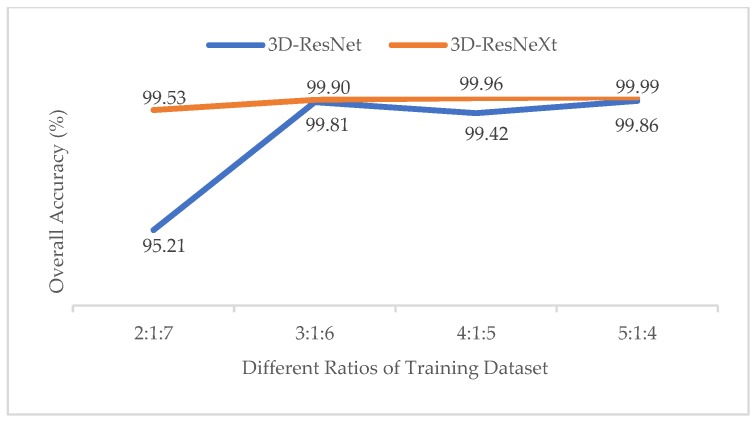
OAs of the 3D-ResNet and 3D-ResNeXt with different ratios of training samples for the KSC dataset.

To test the robustness and generalizability of the proposed 3D-ResNeXt under different ratios of training datasets, the overall accuracies (OAs) of 3D-ResNeXt and 3D-ResNet [34] using different ratios of training datasets are illustrated in Figure 17, Figure 18 and Figure 19. For a small number of training samples such as the ratio 2:1:7 of the IN and KSC datasets, the proposed network generated superior OA and produced a significantly better classification performance than the 3D-ResNet. It means that despite fewer training samples, we can still achieve better classification results by the proposed 3D-ResNeXt based model. With the increasing of the number of training samples, the overall accuracy of the two networks both rises. The OAs of the 3D-ResNet in the three datasets are all lower than that of the proposed network. It can be found that the 3D-ResNeXt network is more suitable for the HSI classification tasks to learn the spectral-spatial features and achieve good classification results than the 3D-ResNet. In summary, it is obvious that the 3D-ResNeXt performs the best than other methods, because instead of extracting spatial or spectral information separately, the 3D-ResNeXt can utilize the spectral-spatial features jointly by 3D-CNN, which means that it can extract more useful and discriminative features than 3D-ResNet and SSRN. In addition, since the proposed network is relatively deeper than SSRN and 3D-ResNet, the 3D-ResNeXt can learn more abstract and relevant spectral-spatial feature if using a larger amount of training samples correspondingly. Therefore, the proposed 3D-ResNeXt model can achieve the best classification accuracies for different training ratios. And with the increase of the ratio of training set, the advantage of our model is more obvious compared with prior models such as 3D-ResNet and SSRN. In summary, our proposed 3D-ResNeXt HSI classification model has strong robustness and stability under different ratios of training datasets.

In the end, we also validated the effectiveness of group convolutions for reducing the number of parameters. In the experiment, we test the number of parameters and the classification performance of 3D-ResNet with different number of residual blocks, and 3D-ResNeXt, which has optimal parameter setting, has the same architecture and the same number of layers with 3D-ResNet. It is clearly shown in Table 20 that the number of parameters of the 3D-ResNeXt is significantly about 3,000,000 less than the 3D-ResNet in the case of the same number of blocks, and our proposed network achieved higher classification accuracies than the 3D-ResNet. When the numbers of blocks of 3D-ResNet and our proposed network increase, the OA indicators in three HSI datasets all decrease a little. Taking the computational cost into consideration, we finally chose the proposed model with four same blocks. It means that we can use a model based on 3D-ResNeXt with fewer parameters, faster convergence, and deeper networks to achieve better performance.

### 3.5. Discussion

The above experiments verify the effectiveness of the 3D-ResNeXt framework in the HSI classification. Compared with the traditional state-of-the-art methods, the end-to-end deep learning methods can extract features automatically, and avoid the complex manual operation and preprocessing of HSI datasets. More importantly, deep learning methods can utilize computational hardware especially GPU efficiently.

Three major differences between our 3D-ResNeXt based HSI classification model and other deep learning-based models are as follows. First, instead of 2D-CNN, the 3D-ResNeXt adopts 3D-CNN that can extract spectral-spatial features and reduce the possibility of the information losing. Group convolutions shows an obvious effectiveness in reducing the number of parameters. Second, the residual connections ensure that the network can be deeper, and the accuracy can be improved. Third, owing to the label smoothing strategy and a method to enrich the information of the input of network, the network can extract more useful and representative features, especially for the classes with a small number of training samples. It should be noted that, our 3D-ResNeXt based model generates high classification accuracy especially for relatively few land-cover categories. Therefore, we did not employ data augmentation to further promote the 3D-ResNeXt performance in current work.

## 4. Conclusion

In this paper, we proposed a supervised 3-D deep learning framework for HSI classification, using the characteristics of the 3D-CNN to extract spectral-spatial features. The designed 3D-ResNeXt HSI classification model alleviated the decreasing-accuracy phenomenon and reduced the number of parameters by using group convolutions and residual connections. Before the input of the main network, we utilized a simple 3D-ResNet network to extract spectral features which are combined with the HSI data processed by dimensionality reduction to enrich the spectrum characteristics of all the classes. Furthermore, label smoothing strategy was used to modify the loss function to improve the classification accuracy of classes with small samples. The experiment results show that the 3D-ResNeXt performs well on the three public datasets with the highest classification accuracy and the most smoothly and accurate maps.

Our 3D-ResNeXt based model can be easily extended to other HSI datasets because of its uniform structural design and deep feature learning capacity. The features can be extracted automatically from input data without any feature engineering. The configuration of hyperparameters depends on the number of training samples and the spatial size of each sample input. The proposed 3D-ResNeXt has much less parameters than the 3D-ResNet while keeping the high accuracy. In addition, in the case of the imbalance of training samples, the classes with small number of training samples may be classified incorrectly by previous methods. The proposed model, which has a spectral-spatial residual architecture, overcomes this difficulty by enriching the spectral information and optimizing the loss function. The above advantages enable our 3D-ResNeXt network to gain a high accuracy in a spectral-spatial way.

From the perspective of feature extraction effectiveness, the future work will focus on the fusion of different deep learning approaches, which can make certain contributions to improving the classification performance.

## Figures and Tables

**Table 1:** Numbers of samples of the IN dataset.

No.	Class	No. of Samples
1	Alfalfa	46
2	Corn-notill	1428
3	Corn-mintill	830
4	Corn	237
5	Grass-pasture	483
6	Grass-trees	730
7	Grass-pasture-mowed	28
8	Hay-windrowed	478
9	Oats	20
10	Soybean-notill	972
11	Soybean-mintill	2455
12	Soybean-clean	593
13	Wheat	205
14	Woods	1265
15	Buildings-Grass-Trees-Drives	386
16	Stone-Steel-Towers	93
Total		10249

**Table 2:** Numbers of samples of the UP dataset.

No.	Class	No. of Samples
1	Asphalt	6631
2	Meadows	18649
3	Gravel	2099
4	Trees	3064
5	Painted metal sheets	1345
6	Bare Soil	5029
7	Bitumen	1330
8	Self-Blocking Bricks	3682
9	Shadows	947
Total		42776

**Table 3:** Numbers of samples of the KSC dataset.

No.	Class	No. of Samples
1	Scrub	761
2	Willow swamp	243
3	CP hammock	256
4	Slash pine	252
5	Oak/Broadleaf	161
6	Hardwood	229
7	Swamp	105
8	Graminoid marsh	431
9	Spartina marsh	520
10	Cattail marsh	404
11	Salt marsh	419
12	Mud flats	503
13	Water	927
Total		5211

**Table 4:** The architecture of the proposed network.

Layer	Output Size	3D-ResNeXt	Connected to
Input	11×11×200		
CONV1	9×9×97, 24	3×3×7, 24 conv s=(1, 1, 2)	Input
CONV2	9×9×97, 32	1×1×7, 32 conv same	CONV1
CONV3	9×9×97, 32	1×1×7, 32 conv same	CONV2
CONV4	9×9×97, 32	3×3×7,32 conv	Input
Add	9×9×97, 32		CONV3, CONV4
Block2_1	9×9×97, 64	3×3×3, 64 conv same	Add
Block2_2	5×5×49, 128	3×3×3, 128 conv s=(2, 2, 2)	Block2_1
Block2_3	3×3×25, 256	3×3×3, 256 conv s=(2, 2, 2)	Block2_2
Block2_4	2×2×13, 512	3×3×3, 512 conv s=(2, 2, 2)	Block2_3
Flatten	26624		Block2_4
Dense1	1024	1024	Flatten
Dense2(SoftMax)	16	16	Dense1

**Table 5:** Training time, test time, and OA under different training dataset ratios on the IN dataset by the proposed method.

Ratios	Training Time (s)	Test Time (s)	OA (%)
2:1:7	2751.91	24.05	99.22
3:1:6	3140.21	20.88	99.82
4:1:5	2709.78	17.41	99.90
5:1:4	3977.16	14.86	**99.96**

**Table 6:** Training time, test time, and OA under different training dataset ratios on the UP dataset by the proposed method.

Ratios	Training Time (s)	Test Time (s)	OA (%)
2:1:7	6077.30	54.85	99.93
3:1:6	7095.93	52.18	99.98
4:1:5	6857.42	39.32	**99.99**
5:1:4	8630.59	40.39	**99.99**

**Table 7:** Training time, test time, and OA under different training dataset ratios on the KSC dataset by the proposed method.

Ratios	Training Time (s)	Test Time (s)	OA (%)
2:1:7	1260.70	10.27	99.53
3:1:6	1384.68	8.66	99.90
4:1:5	1352.76	7.25	99.96
5:1:4	1656.15	5.88	**99.99**

**Table 8:** Training time, test time, and OA for different input spatial sizes on the IN dataset by the proposed method.

Spatial Size	Training Time (s)	Test Time (s)	OA (%)
7×7	1503.56	5.38	99.90
9×9	2173.70	8.75	99.90
11×11	3977.16	14.86	**99.96**
13×13	4721.14	19.41	99.95
15×15	6034.21	23.28	99.90

**Table 9:** Training time, test time, and OA for different input spatial sizes on the UP dataset by the proposed method.

Spatial Size	Training Time (s)	Test Time (s)	OA (%)
7×7	3230.44	18.43	99.93
9×9	4432.76	25.46	99.95
11×11	6857.42	39.32	**99.99**
13×13	9039.73	80.44	**99.99**
15×15	11318.89	78.97	**99.99**

**Table 10:** Training time, test time, and OA for different input spatial sizes on the KSC dataset by the proposed method.

Spatial Size	Training Time (s)	Test Time (s)	OA (%)
7×7	719.82	2.51	99.95
9×9	1045.13	3.87	99.98
11×11	1656.15	5.88	**99.99**
13×13	2278.41	8.72	**99.99**
15×15	2807.50	11.92	**99.99**

**Table 11:** Params, training time, test time, and OA for different cardinality (C) on the IN, UP, and KSC datasets.

Datasets	*C*	Params	Training Time (s)	Test Time (s)	OA (%)
IN	6	21,562,960	2912.32	11.82	99.88
	8	28,825,456	3977.16	14.86	**99.96**
	10	36,130,960	4351.49	15.55	99.90
UP	6	12,118,608	5533.12	31.61	99.98
	8	16,235,376	6857.42	39.32	**99.99**
	10	20,395,152	8077.80	43.65	**99.99**
KSC	6	18,414,160	1337.70	4.87	99.97
	8	24,628,080	1656.15	5.88	**99.99**
	10	30,885,008	1994.49	6.78	99.95

**Table 12:** The Overall (OA) classification accuracy (%) for different methods in three HSI datasets.

Method	SVM	Rank-1 FNN	1D-CNN	2D-CNN-LR	3D-CNN	SSRN	3D-ResNet	3D-ResNeXt
IN	81.67	92.82	87.81	89.99	**99.76**	99.19	99.68	**99.96**
UP	90.58	93.50	92.28	94.04	99.50	99.79	**99.93**	**99.99**
KSC	80.29	95.51	89.23	94.11	99.81	99.61	**99.86**	**99.99**

**Table 13:** Classification results of different methods for the IN dataset.

	3D-CNN	SSRN	3D-ResNet	3D-ResNeXt
OA (%)	**99.76**	99.19	99.68	**99.96**
AA (%)	99.59	98.93	**99.62**	**99.80**
Kappa ×100	**99.72**	99.07	99.64	**99.95**
1	100.0	97.82	100.0	100.0
2	100.0	99.17	99.65	100.0
3	100.0	99.53	99.38	100.0
4	98.94	97.79	97.89	100.0
5	98.95	99.24	98.96	100.0
6	99.30	99.51	100.0	100.0
7	100.0	98.70	100.0	100.0
8	100.0	99.85	100.0	100.0
9	100.0	98.50	100.0	100.0
10	100.0	98.74	100.0	100.0
11	99.69	99.30	100.0	100.0
12	100.0	98.43	98.01	100.0
13	100.0	100.0	100.0	100.0
14	100.0	99.31	100.0	100.0
15	99.37	99.20	100.0	100.0
16	97.22	97.82	100.0	97.22

**Table 14:** Classification results of different methods for the UP dataset.

	3D-CNN	SSRN	3D-ResNet	3D-ResNeXt
OA (%)	99.50	99.79	**99.93**	**99.99**
AA (%)	99.38	99.66	**99.91**	**99.99**
Kappa ×100	99.34	99.72	**99.91**	**99.98**
1	99.67	99.92	99.94	99.98
2	99.89	99.96	100.0	100.0
3	99.80	98.46	100.0	99.98
4	99.87	99.69	99.93	100.0
5	99.71	99.99	99.86	100.0
6	99.52	99.94	99.80	100.0
7	99.26	99.82	100.0	100.0
8	96.73	99.22	99.67	99.98
9	100.0	99.95	100.0	100.0

**Table 15:** Classification results of different methods for the KSC dataset.

	3D-CNN	SSRN	3D-ResNet	3D-ResNeXt
OA (%)	99.81	99.61	**99.86**	**99.99**
AA (%)	99.74	99.33	**99.81**	**99.99**
Kappa ×100	99.79	99.56	**99.84**	**99.99**
1	100.0	99.70	100.0	100.0
2	100.0	99.88	100.0	100.0
3	100.0	99.00	99.01	100.0
4	97.25	98.26	99.06	99.99
5	100.0	99.03	100.0	100.0
6	100.0	99.43	100.0	100.0
7	100.0	97.03	100.0	100.0
8	99.42	99.54	100.0	100.0
9	100.0	99.70	99.52	100.0
10	100.0	99.96	100.0	100.0
11	100.0	99.80	100.0	100.0
12	100.0	100.0	100.0	100.0
13	100.0	100.0	100.0	100.0

**Table 16:** Training time and test time for different networks in the IN dataset.

Method	Training Time (s)	Test Time (s)
3D-CNN	1157.71	4.19
SSRN	767.33	3.53
3D-ResNet	2604.53	9.99
3D-ResNeXt	3977.16	14.86

**Table 17:** Training time and test time for different networks in the UP dataset.

Method	Training Time (s)	Test Time (s)
3D-CNN	1872.98	12.53
SSRN	1368.08	12.45
3D-ResNet	5042.26	28.39
3D-ResNeXt	6857.42	39.32

**Table 18:** Training time and test time for different networks in the KSC dataset.

Method	Training Time (s)	Test Time (s)
3D-CNN	535.18	2.05
SSRN	386.14	1.73
3D-ResNet	1207.17	4.24
3D-ResNeXt	1656.15	5.88

**Table 19:** Classification results of the 3D-ResNeXt with different loss functions on the IN, UP, and KSC datasets.

		3D-ResNeXt (Cross-Entropy)	3D-ResNeXt (with Label Smoothing)
IN	OA (%)	99.83	**99.96**
	AA (%)	99.70	**99.80**
	Kappa ×100	99.81	**99.95**
UP	OA (%)	99.93	**99.99**
	AA (%)	99.91	**99.99**
	Kappa ×100	99.91	**99.98**
KSC	OA (%)	99.71	**99.99**
	AA (%)	99.76	**99.99**
	Kappa ×100	99.68	**99.99**

**Table 20:** Comparison on params, training time, test time, and OA between the 3D-ResNet and our 3D-ResNeXt with different number of blocks on the IN, UP, and KSC datasets.

Datasets	Method	Params	Training Time (s)	Test Time (s)	OA (%)
IN	3D-ResNet-4	32,176,496	2604.53	9.99	99.68
	3D-ResNet-6	34,472,560	5230.70	19.42	99.29
	3D-ResNeXt-4	**28,825,456**	3977.16	14.86	**99.96**
	3D-ResNeXt-6	**29,268,080**	5957.54	22.09	**99.99**
UP	3D-ResNet-4	19,586,416	5042.26	28.39	99.93
	3D-ResNet-6	21,882,480	9582.73	54.62	99.92
	3D-ResNeXt-4	**16,235,376**	6857.42	39.32	**99.99**
	3D-ResNeXt-6	**16,678,000**	11088.90	64.23	**99.98**
KSC	3D-ResNet-4	27,979,120	1207.17	4.24	99.86
	3D-ResNet-6	30,275,184	2402.87	8.72	99.62
	3D-ResNeXt-4	**24,628,080**	1656.15	5.88	**99.99**
	3D-ResNeXt-6	**25,070,704**	2707.75	9.82	**99.96**
